# LASIK following Small Incision Lenticule Extraction (SMILE) Lenticule Re-Implantation: A Feasibility Study of a Novel Method for Treatment of Presbyopia

**DOI:** 10.1371/journal.pone.0083046

**Published:** 2013-12-11

**Authors:** Chris H. L. Lim, Andri K. Riau, Nyein C. Lwin, Shyam S. Chaurasia, Donald T. Tan, Jodhbir S. Mehta

**Affiliations:** 1 Faculty of Medicine, University of New South Wales, Sydney, New South Wales, Australia; 2 Tissue Engineering and Stem Cell Group, Singapore Eye Research Institute, Singapore, Singapore; 3 Singapore National Eye Centre, Singapore, Singapore; 4 Department of Ophthalmology, Yong Loo Lin School of Medicine, National University of Singapore, Singapore, Singapore; 5 SRP Neuroscience and Behavioral Disorders, Duke-NUS Graduate Medical School, Singapore, Singapore; 6 Department of Clinical Sciences, Duke-NUS Graduate Medical School, Singapore, Singapore; Bascom Palmer Eye Institute, University of Miami School of Medicine, United States of America

## Abstract

Presbyopia remains a major visual impairment for patients, who have previously undergone laser refractive correction and enjoyed unaided distance vision prior to the onset of presbyopia. Corneal stromal volume restoration through small incision lenticule extraction (SMILE) lenticule re-implantation presents an opportunity for restoring the patients’ non-dominant eye to previous low myopia to achieve a monovision. In this study, we investigated the feasibility of performing LASIK after lenticule re-implantation as a method to create presbyopic monovision. A -6.00D SMILE correction was performed in 9 rabbit eyes. The lenticules were cryopreserved for 14 days and re-implanted. Five weeks later, 3 of these eyes underwent LASIK for -5.00D correction (RL group); 3 underwent LASIK flap creation, which was not lifted (RN); and no further procedures were performed on the remaining 3 eyes. These groups were compared with 3 eyes that underwent standard LASIK for a -5.00D correction (LO); 3 that underwent creation of non-lifted flap (LN); and 3 non-operated eyes. Rabbits were euthanized 1 day post-surgery. Tissue responses were analyzed by immunohistochemistry, slit lamp and in vivo confocal microscopy (IVCM). Intrastromal irregularities and elevated reflectivity levels of the excimer-ablated plane were observed on slit lamp and IVCM, respectively in the RL group. The results were comparable (P = 0.310) to IVCM findings in the LO group. RL and LO groups showed similar fibronectin expression levels, number of CD11b-positive cells (P = 0.304) and apoptotic cells (P = 0.198). There was no difference between the RN and LN groups in reflectivity levels (P = 0.627), fibronectin expression levels, CD11b-positive cells (P = 0.135) and apoptotic cells (P = 0.128). LASIK can be performed following lenticule re-implantation to create presbyopic monovision. The tissue responses elicited after performing LASIK on corneas that have undergone SMILE and subsequent lenticule re-implantation are similar to primary procedure.

## Introduction

Laser in-situ keratomileusis (LASIK) is one of the most widely performed elective surgical procedure worldwide [1,2]. Its popularity stems from the short procedure time, excellent visual outcomes, pain-free and rapid post-operative visual recovery, as well as established safety profile [3,4]. This procedure first involves the creation of a corneal flap with either a mechanical microkeratome, or more recently, a femtosecond laser (FSL) [5-8]. The resulting flap is reflected and the exposed underlying stroma is then ablated with an excimer laser before the flap is repositioned. However, LASIK is not without its post-operative complications, which includes dry eye syndrome [9], transient light sensitivity [10], and keratectasia [11]. 

There has been a growing level of interest in recent years surrounding the application of FSL only surgery for myopic and astigmatic correction. FSLs emit ultra-short light pulses at high frequencies, permitting photodisruption of corneal tissue at lower energy levels [12,13]. This is accomplished by initiating rapid gas expansion, which leads to cavitation bubbles formation, thereby creating an intrastromal incision plane with minimal heat development [13]. The photodisruption process, therefore, results in minimal collateral damage to adjacent tissues compared to the traditionally high-energy excimer lasers utilized in LASIK [14]. 

The use of FSL technology in refractive surgery has been largely restricted to the creation of corneal flaps, while the actual refractive correction remains the domain of the excimer laser. With the advancement of the technology, FSL is now capable of correcting the refractive error by sculpting an intrastromal refractive lenticule, which is then removed by the surgeon. The technique, coined as refractive lenticule extraction (ReLEx), can be performed in 2 ways. The earlier variant of ReLEx, femtosecond lenticule extraction (FLEx), involves the creation of a flap akin to a LASIK flap [14-16]. The flap is subsequently lifted, thereby exposing the refractive lenticule, which is then stripped away. The lenticule extraction technique has been further refined into a small incision technique (SMILE, small incision lenticule extraction) [17], which involves the creation of an arcuate incision of ≤3mm in width, through which the refractive lenticule is extracted. By obviating the creation of a flap, the SMILE procedure may minimize disruption to the sub-basal nerve plexus, possibly reducing post-operative dry eye symptoms [18], and also eliminating the risk of flap dislocation. 

A refractive lenticule is the immediate by-product of ReLEx in all cases. The feasibility of cryopreservation and subsequent re-implantation of the extracted refractive lenticule to correct the original myopic correction has been previously proposed [19-21]. This ability to restore corneal stromal volume by lenticule re-implantation and improve upon the post-refractive surgical biomechanical strength of the cornea has numerous possible applications; one of them being the unique opportunity to perform a secondary refractive surgical procedure, i.e. LASIK. This may be performed following refractive lenticule re-implantation, in order to achieve a monovision correction in patients with presbyopia who have previously undergone ReLEx for myopic correction. A monovision is achieved when the dominant eye is corrected for emmetropia, while myopia is either deliberately undercorrected or induced in the non-dominant eye [22]. Although patients will experience a reduction in distance vision and stereopsis from the resultant anisometropia, ametropia of the non-dominant eye increases the functional zone of near and intermediate vision, as long as the difference in refractive correction between both eyes is not too significant. 

In this study, we demonstrate the feasibility of performing LASIK following reversal of myopic ReLEx through refractive lenticule re-implantation, as a novel method of inducing monovision in a rabbit model of SMILE. In brief, the eyes underwent a -6.00D spherical correction with SMILE and reversal of the myopic correction 14 days later. Once sufficient corneal wound healing was allowed to take place over 5 weeks, a LASIK flap was created anterior to the re-implanted lenticular anterior interface and a -5.00D spherical correction performed with an excimer laser to simulate monovision correction by creating a 1.00D difference between both eyes. 

## Methods

### Animals

**Figure 1:**
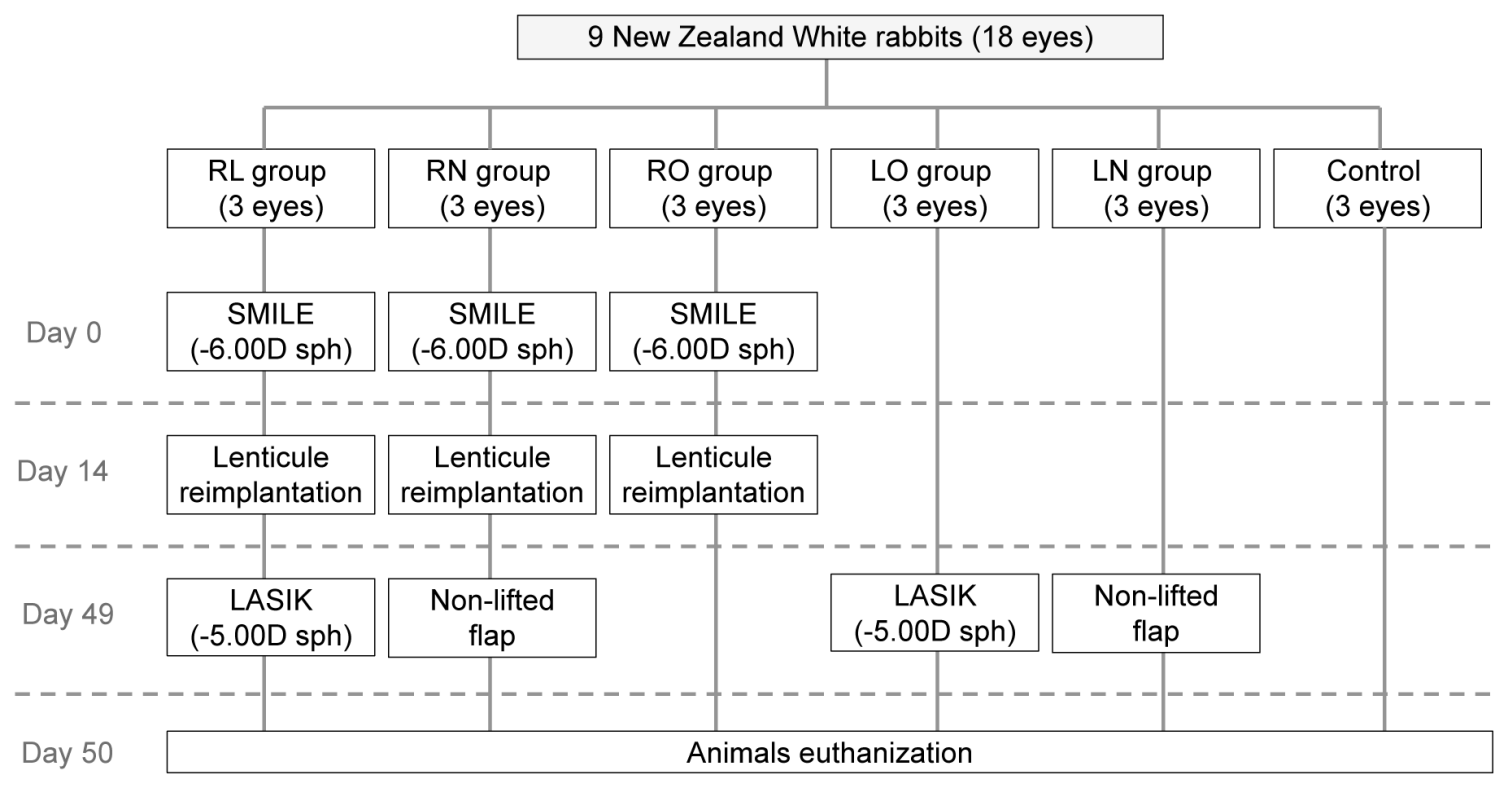
Treatment allocation of rabbits’ eyes in the study. On day 0, small incision lenticule extraction (SMILE) was performed to induce a refractive spherical correction (sph) of -6.00D in nine eyes. The extracted lenticules were cryopreserved for 14 days and autologous re-implantation was performed thereafter. Five weeks later, the 9 eyes were divided into three different groups: three eyes had a corneal flap created, which was not lifted (this group is abbreviated as RN); while another three eyes underwent LASIK for -5.00D spherical correction (RL); and no further procedures were performed on the remaining three eyes (RO). In the remaining 9 eyes that did not undergo SMILE on day 0, 3 eyes underwent LASIK for a -5.00D refractive spherical correction (LO); while another 3 eyes underwent the creation of a non-lifted LASIK flap (LN). The last 3 non-operated eyes served as controls.

Nine 12 to 15-week-old New Zealand White rabbits (3-4kg in body weight) were procured from the National University of Singapore. SMILE was performed to induce a refractive spherical correction of -6.00D in nine eyes. The extracted stromal lenticules were stored at -80°C for 14 days and autologous re-implantation was performed thereafter. Five weeks later, the nine eyes were further divided into three different groups: three eyes had a corneal flap created, which was not lifted (this group is abbreviated as RN); while another three eyes underwent LASIK for -5.00D spherical correction (RL). No further procedures were performed on the remaining three eyes (RO). In the remaining 9 eyes that did not undergo SMILE, 3 eyes underwent LASIK for a -5.00D refractive spherical correction (LO), while another 3 eyes underwent the creation of a non-lifted LASIK flap (LN). The last 3 non-operated eyes served as controls for this study. None of the rabbits were subjected to binocular visual sensory deprivation at any point during the study. All rabbits were euthanized 1 day post-operatively. The treatment allocation of rabbit eyes in this study has been illustrated in Figure 1.

During the surgical procedures, and pre- and post-operative examination, the rabbits were anesthetized with xylazine hydrochloride (5 mg/kg intramuscularly; Troy Laboratories, Smithfield, Australia) and ketamine hydrochloride (50 mg/kg intramuscularly; Parnell Laboratories, Alexandria, Australia). Euthanasia was performed following the administration of anaesthesia via an intracardiac bolus injection of sodium pentobarbital (Jurox, Rutherford, Australia). All animals were handled according to the guidelines outlined in the ARVO Statement for the Use of Animals in Ophthalmic and Vision Research. Ethics approval for this study protocol was sought and obtained from the Institutional Animal Care and Use Committee of SingHealth (Singapore).

### Small incision lenticule extraction (SMILE) procedure

SMILE was performed using a VisuMax femtosecond laser system (VisuMax, Carl Zeiss Meditec, Jena, Germany). A small interface cone was used in all of the procedures. Once suction was applied, laser incisions were made in the following automated sequence: a spiral in pattern for the posterior surface of the lenticule, spiral out pattern for the anterior surface of the lenticule [23], followed by a superiorly placed 3mm wide incision. The diameter and depth of the cap were set at 7.5 mm and 130 μm, respectively. The diameter of the lenticule (equating to the optical zone) was 6.5 mm. The following FSL parameters were used: the power settings were set to 200nJ in the creation of the lenticule, lenticule side, cap and cap-side cuts. A side cut of 90° was used for this study. The spot distance and tracking spacing settings were set at 3 μm/ 3 μm for lenticule, 2 μm/ 2 μm for lenticule side, 3 μm/ 3 μm for cap, and 2 μm/ 2 μm for cap side. 

After the laser sequence was completed, a Seibel spatula (Rhein Medical Inc., Petersburg, FL) was inserted into the superiorly placed 3mm incision to gain access to the intrastromal lenticule. Following which, a proprietary lamellar dissector (Asico, Westmont, IL), which we designed for SMILE lamellar dissection, was introduced to dissect microadhesions; firstly on the anterior and then the posterior surfaces of the lenticule from the surrounding stroma. Once these planes were separated, the lenticule was extracted using a co-axial Tan DSAEK forceps (Asico). The cornea stromal pocket was then irrigated with balanced salt solution via a 24-gauge cannula. 

### Stromal lenticule storage and re-implantation

The extracted stromal lenticules were placed on rigid gas permeable (RGP) contact lenses (Bausch and Lomb, Rochester, NY) and cryopreserved as previously described [19-22]. Due care was given to the anatomical orientation of the stromal lenticules while transferring these lenticules onto RGP lenses for cryopreservation. A marking was made on the RGP lenses at the 12 o'clock position to indicate the anatomically equivalent aspect of the stromal lenticule within the cornea. The RGP contact lenses with the refractive lenticules were placed in contact lens cases with the wells filled with a stock freezing solution containing 10% FBS (fetal bovine serum; Sigma, St. Louis, MO) and 20% dimethyl sulfoxide (DMSO; Sigma). Freezing of the RGP contact lenses and contact lens case containing the lenticule was carried out at a controlled cooling rate within a cryo-container (“Mr. Frosty”, Thermo Fisher Scientific, Roskilde, Denmark) in a -80°C freezer overnight, and transferred into liquid nitrogen the following day for long-term storage.

Re-implantation of the refractive lenticule was performed 14 days after the initial SMILE procedure. A day before the procedure, the contact lens cases were first retrieved from storage and transferred to -80°C freezer overnight. Two hours before re-implantation, the lenticules were retrieved from the freezer and allowed to warm to room temperature. The refractive lenticules were removed from the stock freezing solution and rinsed thoroughly with balanced salt solution. Following this, the rabbits were anaesthetised and a Sinskey hook (Rhein Medical Inc.) was used to open the previously created 3-mm arcuate incision. A lamellar dissector (Asico) was then inserted through the small incision to gently release the cap-stromal bed adhesions. The refractive lenticule was partially folded and reintroduced into the original site through the small incision using a pair of corneal forceps by carefully observing the 12 o’clock orientation of the lenticule on the stromal bed. Thereafter, the lamellar dissector was used to spread the refractive lenticule out within the stromal pocket. 1 mL of gentamicin sulphate (40 mg/ml; Shin Poong Pharmaceutical, Seoul, South Korea) and dexamethasone sodium phosphate (40 mg/ml; Shin Poong Pharmaceutical, Seoul, South Korea) were each administered via a subconjunctival injection before two interrupted sutures were used to close the incision site. Prednisolone acetate (1%; Allergan, Irvine, CA) and tobramycin (0.3%; Alcon) drops were also administered four times daily for the duration of one week. 

### Femtosecond laser-assisted LASIK procedure

LASIK flaps were created with the VisuMax femtosecond laser system (Carl Zeiss Meditec) as previously described [14]. The following laser settings were used for rabbit eyes in the RN and RL groups: 80 µm flap thickness; 7.9 mm flap diameter; 170 nJ power; and spot distance and tracking spacing of 4.8 µm/4.8 µm for lamellar and 2 µm/2 µm for flap side cuts, respectively. All laser parameters were unchanged in the LO and LN groups, except for the flap thickness, which was set at 130 µm. The corneal flap of rabbit eyes in the RL and LO groups were lifted and a 6.5-mm optical zone ablation was performed using an excimer laser (Technolas; Bausch & Lomb) with the following parameters: spot size 2.0 mm diameter, fluence 120 mJ/cm^2^, and repetition rate 50 Hz. The flap was subsequently repositioned and an interrupted suture used to hold the flap in place. A bandage contact lens was immediately applied and temporary tarsorrhaphy was used to close the eyelids of eyes with the lifted flaps using a 6-0 silk suture. 

### Corneal imaging: Slit lamp photography and anterior segment-optical coherence tomography (AS-OCT)

Slit-lamp (Zoom Slit Lamp NS-2D; Righton, Tokyo, Japan) and AS-OCT (RTVue Fourier-Domain OCT; Optovue, Fremont, CA) photographs were captured at the following time intervals: before any surgical procedure throughout the study; 1 week and 2 weeks after SMILE; 1 week, 2 weeks, 3 weeks and 5 weeks post-re-implantation of the refractive lenticule; and 1 day after the secondary laser refractive surgery, LASIK, in the RL group. Images were also taken before surgery and 1 day after RN, LO and LN procedures. For AS-OCT, the examiner adjusted the system to position the vertex at the center of the image and then slowly moved the AS-OCT away from the cornea until the vertical white reflection was barely visible. Measurements of central corneal thickness were obtained at the center (0.0 mm) and at 1 mm either side of the centre (+1.0 mm, -1.0 mm). The mean value of the three readings was then reported.

### In vivo confocal microscopy

In vivo confocal microscopy (HRT3; Heidelberg Engineering GmbH, Heidelberg, Germany) was performed before any surgical procedure throughout the study; at 1 week and 2 weeks after SMILE; 1 week, 2 weeks, 3 weeks and 5 weeks after re-implantation of the refractive lenticule; and 1 day after the secondary laser refractive surgery, LASIK, in the RL group. En-face images were also taken before surgery and 1 day after RN, LO and LN procedures. A carbomer gel (Vidisic; Mann Pharma, Berlin, Germany) was used as the immersion fluid. The central aspects of the corneas were examined with a minimum of 3 z-axis scans, consisting of the entire corneal thickness. These micrographs were analysed and the lenticule-stromal interfaces identified. Semi-quantitative analysis of the flap interface reflectivity was performed using Image J (developed by Wayne Rasband, National Institutes of Health, Bethesda, MD; available at http://rsb.info.nih.gov/ij/index.html) by measuring the mean gray value of the reflective particles. 

### Tissue fixation and sectioning

After the rabbits were euthanized, the eyes were enucleated and the corneas excised. These corneas were embedded in an optimum cutting temperature (OCT) cryo-compound (Leica Microsystems, Nussloch, Germany) and stored at -80°C until sectioning. Using a cryostat (Microm HM550; Microm, Walldorf, Germany), 8 μm serial sagittal corneal sections were cut and placed on polylysine-coated glass slides. These slides were stored at -80°C until immunofluorescent staining was performed. 

### Immunofluorescent staining

The sections were air dried and fixed with 4% paraformaldehyde (Sigma, St. Louis, MO). Following which, they were washed with 1X PBS, and incubated in 1X PBS, 0.15% Triton X-100 (Sigma) to increase cellular permeability. These slides were then incubated in 4% bovine serum albumin (Sigma), a blocking reagent, and incubated overnight at 4°C with primary antibodies thereafter. The antibodies used were either mouse monoclonal antibody against cellular fibronectin (Millipore, Billerica, MA) with a dilution factor of 1:400, mouse monoclonal antibody against CD11b (BD Pharmingen, Franklin Lakes, NJ) with a dilution factor of 1:100, or prediluted mouse monoclonal antibody (Invitrogen, Carlsbad, CA) against Ki-67. After washing the slides with 1X PBS the next day, the sections were incubated with a goat anti-mouse Alexa Fluor 488-conjugated secondary antibody (Invitrogen) for 1 hour. The slides were then washed with 1X PBS before being mounted with a medium containing DAPI (UltraCruz Mounting Medium; Santa Cruz Biotechnology, Santa Cruz, CA). The sections were visualized and images captured using a fluorescence microscope (Zeiss AxioImager Z1; Carl Zeiss, Oberkochen, Germany).

### TUNEL Assay

A fluorescence-based TUNEL assay (In Situ Cell Death Detection Kit; Roche Applied Science, Indianapolis, IN) was used to detect apoptotic cells in the sections. This assay was performed according to the manufacturer’s instructions. 

### Statistical Analysis

Data were expressed as mean ± standard deviation (SD). The P value was determined using the Mann-Whitney U test. A value of P<0.05 was considered to be statistically significant. All statistical analysis was performed using SPSS software (version 21.0, SPSS Inc., Chicago, IL). 

## Results

### Slit lamp photography

**Figure 2:**
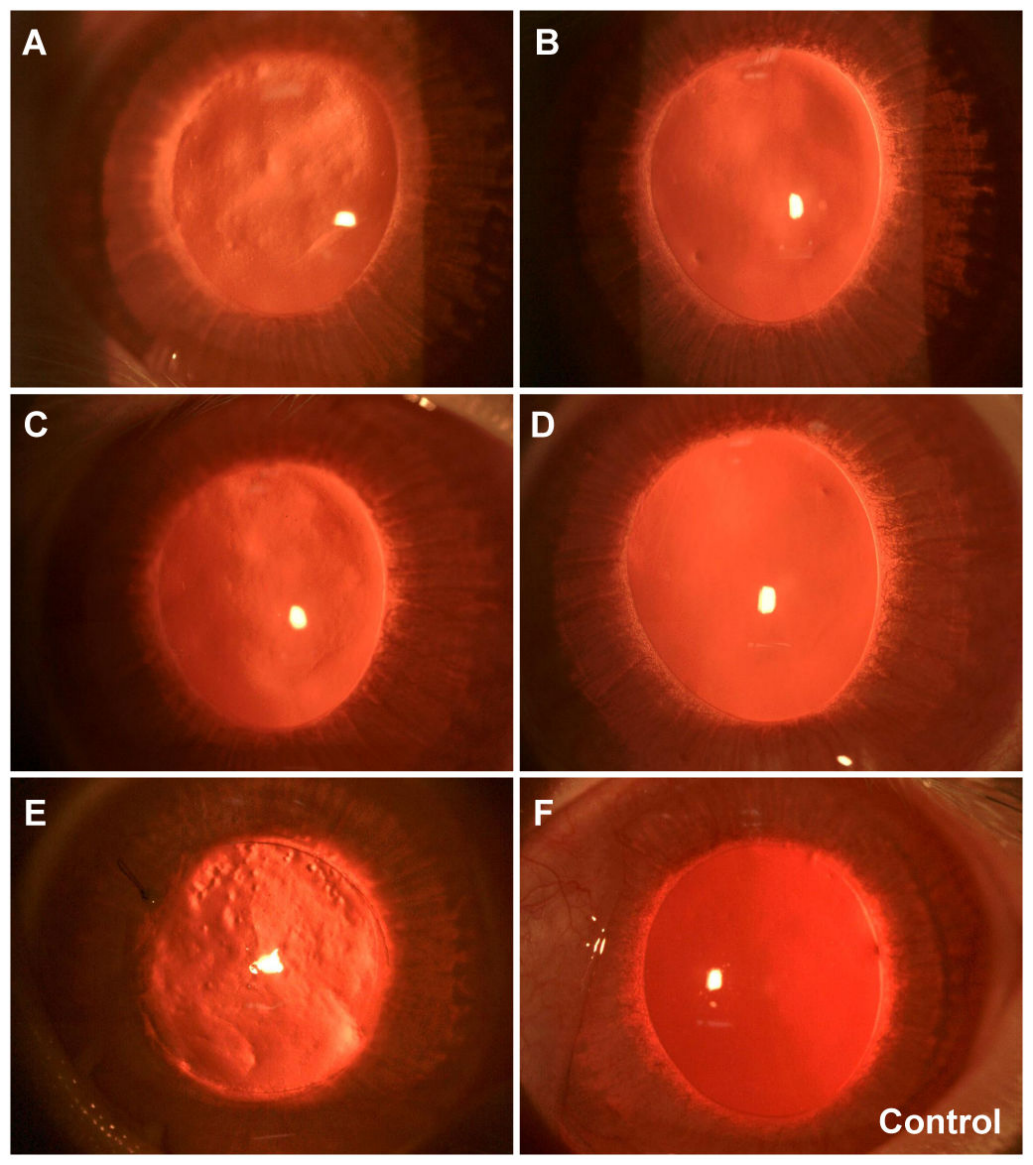
Slit lamp microscopy images of corneas following refractive lenticule re-implantation and subsequent LASIK to induce monovision. Intrastromal irregularities were evident on retro-illumination photographs on week 1 post-re-implantation (A), but appeared to gradually resolve from week 2 (B) to week 3 (C) and stabilized by week 5 (D) post-re-implantation. (E) Slit lamp photograph of the cornea on day 1 post-LASIK demonstrating the presence of intrastromal irregularities at the laser keratotomy site. Interrupted sutures used to hold the flap in place were visible in the 10 o’clock position. (F) Image of a cornea from the control group.

The slit lamp photographs (Figure 2A-E) are representative corneal images from one of the rabbits in the RL group. Retro-illumination photography demonstrated continual improvement in corneal clarity over time following re-implantation of the refractive lenticule (Figure 2A-D). No complications such as diffuse lamellar keratitis were noted in all cases. Presence of irregularities within the corneal stroma was noted in the first week following re-implantation (Figure 2A). These irregularities gradually reduced following suture removal, and disappeared by the second week (Figure 2B). By week 5, the corneas were comparable to those in the control group in terms of clarity (Figure 2D and F). On examination of the corneas 1 day following LASIK, the presence of intrastromal irregularities in areas where excimer ablation was performed could be observed (Figure 2E). The flap side cut was also visible at this time point.

### Cross-sectional visualization by AS-OCT

**Figure 3:**
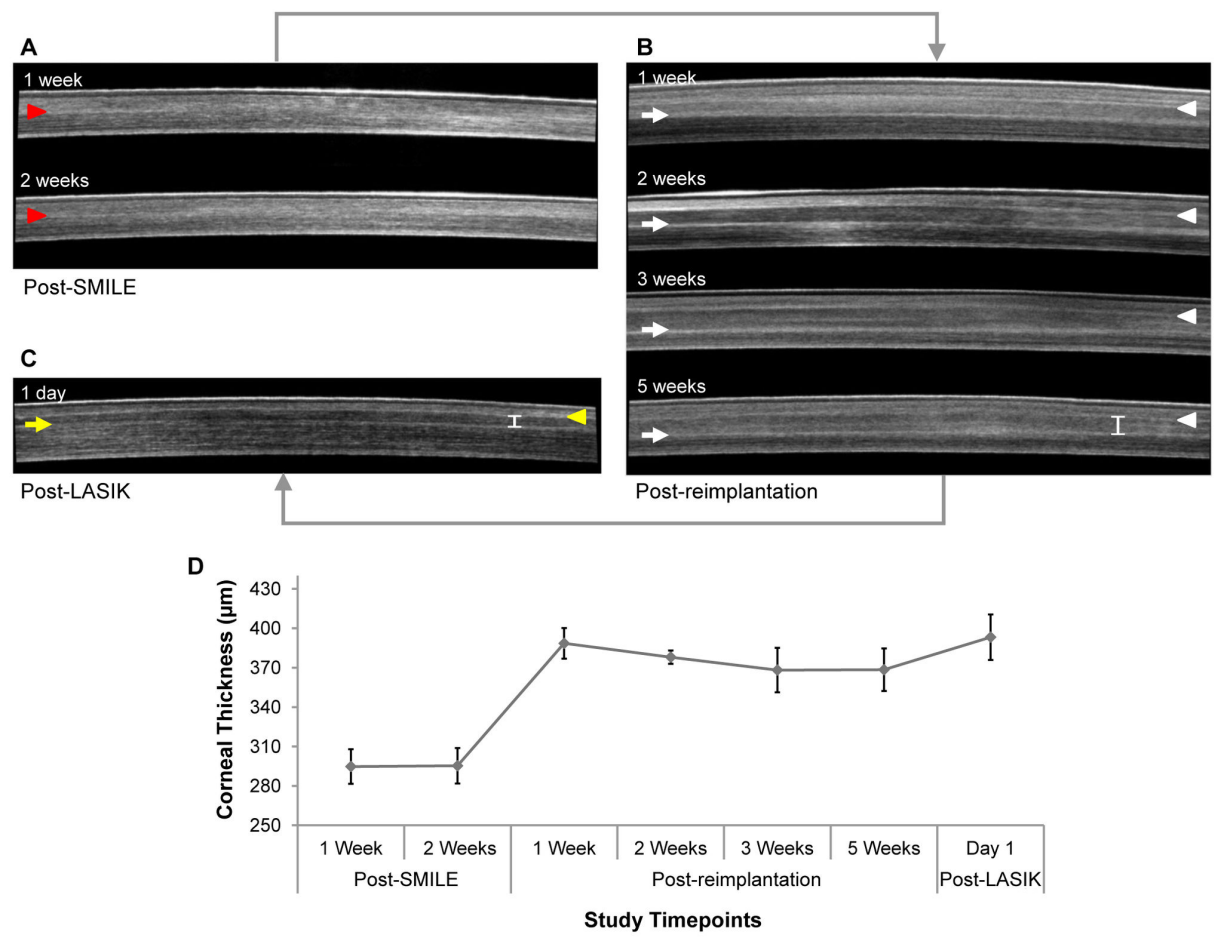
Anterior segment-optical coherence tomography (AS-OCT) images of the central cornea at various study time points. The AS-OCT images (A-C) are representative corneal cross-sectional images from one of the rabbits that underwent lenticule re-implantation and subsequent LASIK to induce monovision. The reduction in corneal thickness following SMILE (A) was evident, when compared to images of the cornea following refractive lenticule re-implantation (B). The bottom left panel (C) contains an image taken 1-day post-LASIK. (D) A graph depicting the corneal thickness measured on AS-OCT images at various time points. Height of error bars represents standard deviation. Red arrowheads indicate the SMILE cap-stromal bed interface. White arrowheads and arrows indicate the anterior and posterior planes of the re-implanted lenticule, respectively. Yellow arrowhead indicates the excimer laser ablated stromal plane and yellow arrow marks the posterior interface of the re-implanted lenticule. A caliper in (B) and (C) is used to show the thinner lenticule within the cornea resulted from excimer laser ablation (C) compared to the cornea before being subjected to LASIK (D).

The AS-OCT images (Figure 3A-C) are representative corneal cross-sectional images from one of the rabbits in the RL group. The corneal cap-stromal bed interface was barely visible (indicated by red arrowhead) in post-SMILE corneas (Figure 3A). However, the anterior (indicated by white arrowhead) and posterior interfaces (indicated by white arrow) between the lenticule and stroma were clearly visible after lenticule re-implantation on AS-OCT imaging over 5-week follow-up period (Figure 3B). Thickening of the corneas relative to post-SMILE corneal thickness following re-implantation was noted (Figure 3A, B and D), indicative of stromal volume restoration. The corneas were relatively thicker 1-week post-re-implantation compared to subsequent follow-up examinations, attributable to the presence of corneal edema, which gradually resolved over serial AS-OCT examinations. An increase in corneal thickness relative to post-re-implantation corneas was noted 1-day post-LASIK (Figure 3B, C and D). This, once again, most likely occurred as a result of the corneal inflammatory response and edema following LASIK. Additionally, the re-implanted lenticule also appeared thinner after excimer laser ablation of the anterior stroma (Figure 3C), indicated by the shorter caliper than that annotated in post-operative week 5 corneal AS-OCT image in Figure 3B.

### In vivo confocal microscopy

**Figure 4:**
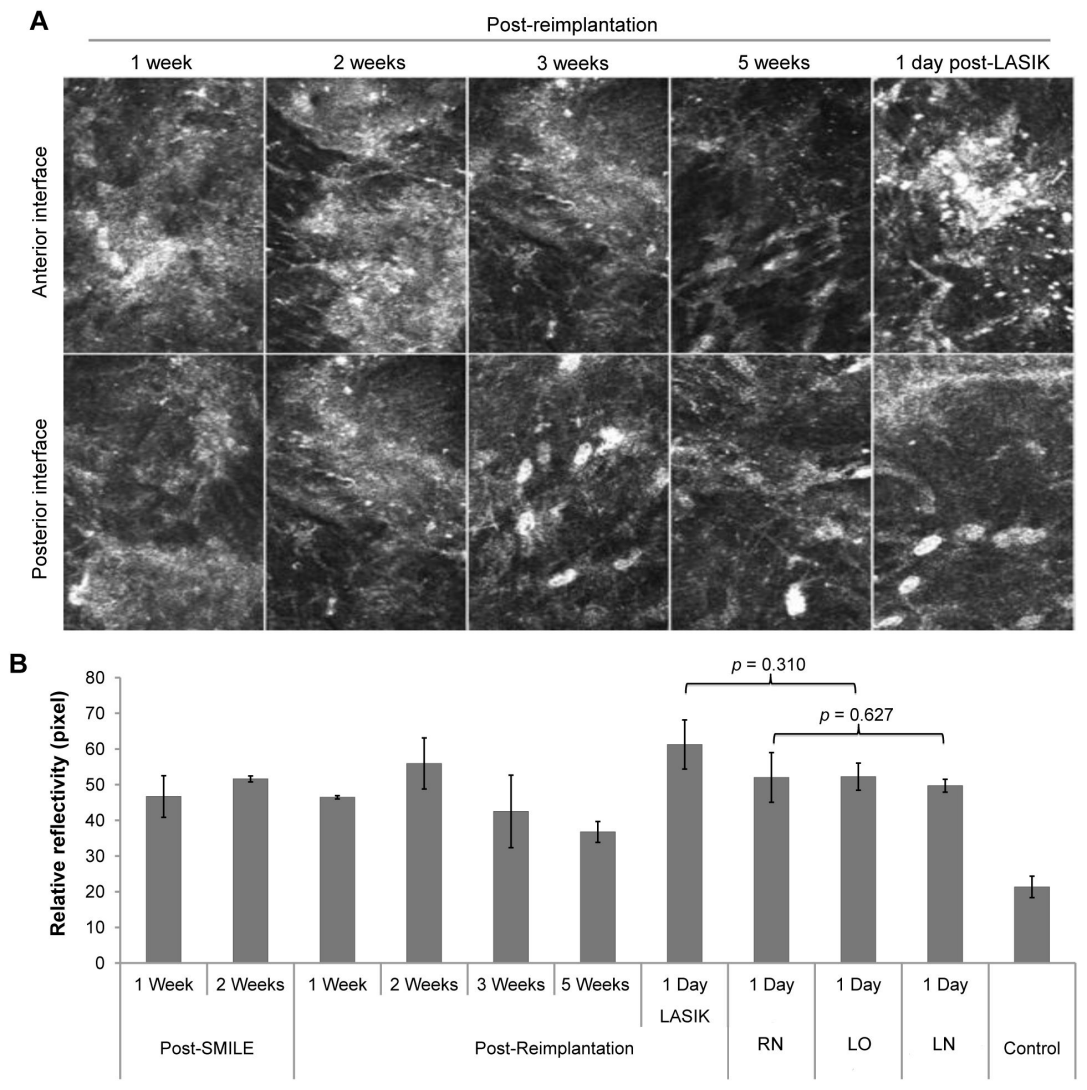
In-vivo confocal micrographs depicting corneal stroma at various time points following refractive lenticule re-implantation and 1 day post-LASIK. The images (A) are representative corneal en-face images from one of the rabbits that underwent lenticule re-implantation and subsequent LASIK to induce monovision. (A) Top row shows images of the anterior border of the reimplanted lenticule and excimer laser ablation plane in cornea that underwent LASIK following lenticule re-implantation. Bottom panel consists of micrographs of the posterior border of the reimplanted lenticule. Increased reflectance and acellularity in anterior and posterior planes were observed 1 week after re-implantation. Small particles of varying sizes were seen at both interfaces of the reimplanted lenticules, which could be attributed to inflammatory cells or surgical debris. However, the haziness and particles gradually resolved over time and keratocyte re-population could be seen as early as 3 weeks post-re-implantation. Following LASIK, a highly reflective and acellular layer with interspersed particles was observed at the anterior interface, which was now the excimer laser ablated plane. (B) Bar graph depicting the relative reflectivity levels at the anterior reflective interface following various interventions and at various time points in the study. Error bars represent standard deviation. RN = corneas that underwent lenticule re-implantation and subsequent creation of a LASIK flap, which was not lifted. LO = corneas that underwent LASIK only. LN = corneas that underwent creation of a LASIK flap, which was not lifted.

The in vivo confocal micrographs (Figure 4A) are representative corneal en-face images from one of the rabbits in RL group. We were able to visualize the anterior (top panel) and posterior (bottom panel) stromal-lenticular interfaces on in vivo confocal microscopy (Figure 4A). Increased reflectance and acellularity in both planes were observed 1 week after re-implantation. Small particles of varying sizes were also seen at the anterior and posterior interfaces of the reimplanted lenticules, which could be attributed to inflammatory cells. However, these features gradually resolved over the follow-up period and keratocyte re-population could be seen as early as 3 weeks post-re-implantation. Following LASIK, a highly reflective and acellular layer with interspersed particles was observed at the anterior interface, which was the excimer laser ablated stromal plane. 

The relative reflectivity levels of the anterior reflective interface obtained from all experimental groups were plotted in a bar graph (Figure 4B). No statistically significant differences in the reflectivity level of interface were noted between post-RL and post-LO groups (P=0.310), and between post-RN and post-LN groups (P=0.627).

### Immunohistochemical analysis

**Figure 5:**
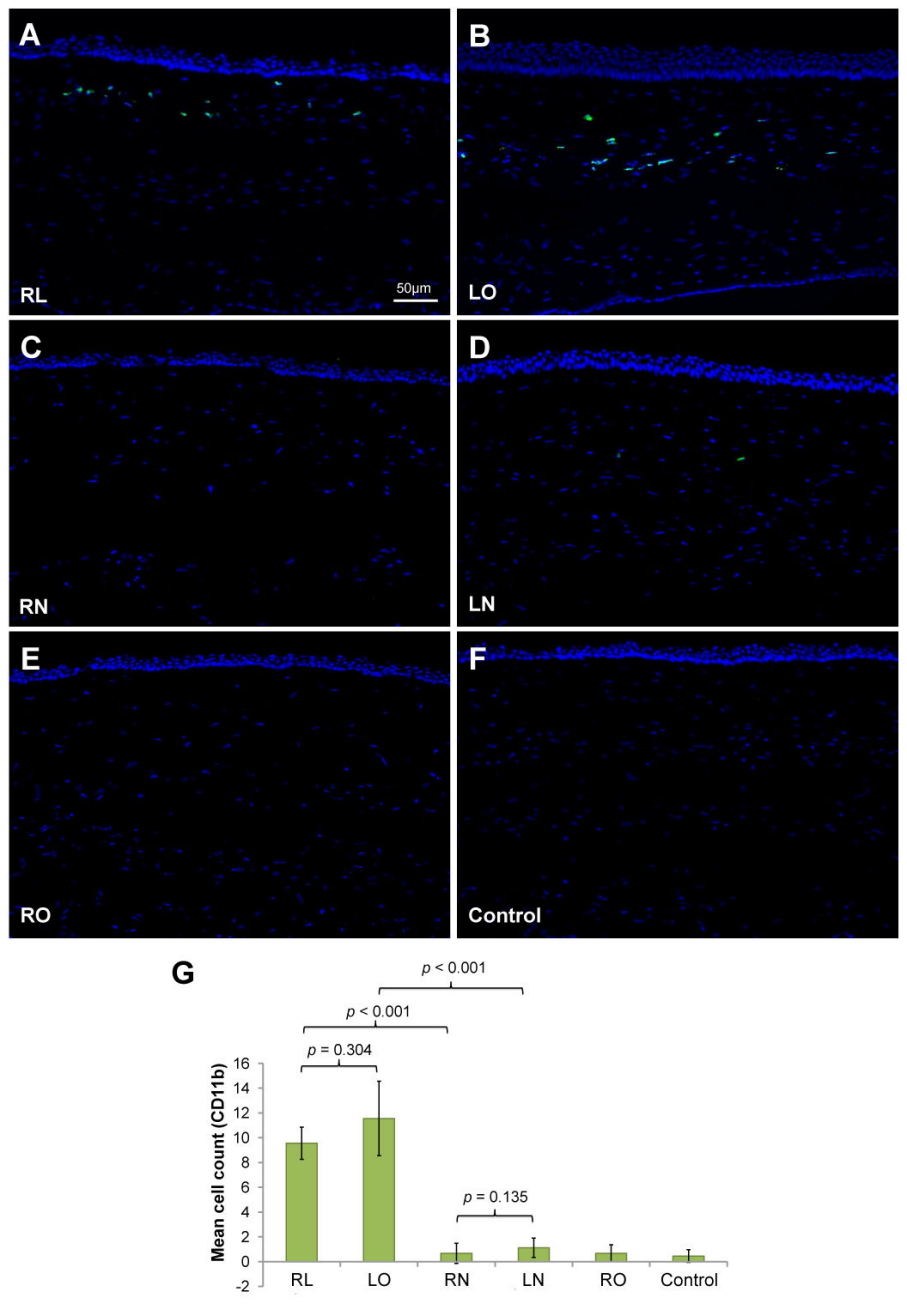
Inflammatory cells in the central cornea detected by CD11b immunostaining. (A) CD11b was expressed along the excimer laser ablation plane in corneas that underwent lenticule re-implantation and subsequent LASIK (RL group) and also in (B) corneas that underwent LASIK only (LO). (C) No CD11b-positive cells were observed in corneas that underwent lenticule re-implantation and subsequent creation of a LASIK flap, which was not lifted (RN), (D) corneas that underwent LASIK flap creation, which was left intact (LN), (E) corneas that underwent lenticule re-implantation only (RO), as well as (F) non-operated corneas (control). All images were captured at a magnification of 100x. (G) Mean cell counts in the central cornea stroma following CD11b staining. Error bars represent standard deviation.

Abundant CD11b expressing cells were detected along the excimer laser ablated plane in the central corneal stroma of the RL and LO groups (Figure 5A and B). Only a few CD11b-positive cells were seen along the non-lifted flap interface in the RN (1.5 ± 0.9) and LN (1.1 ± 0.8) groups (Figure 5C and D). CD11b staining was absent in the RO and control groups (Figure 5E and F). No differences were noted in the mean CD11b cell count between the RL and LO groups (P=0.304), and between the RN and LN groups (P=0.135). Statistically significant differences were found between the RL and RN groups (P<0.001), and between the LO and LN groups (P<0.001) (Figure 5G).

**Figure 6:**
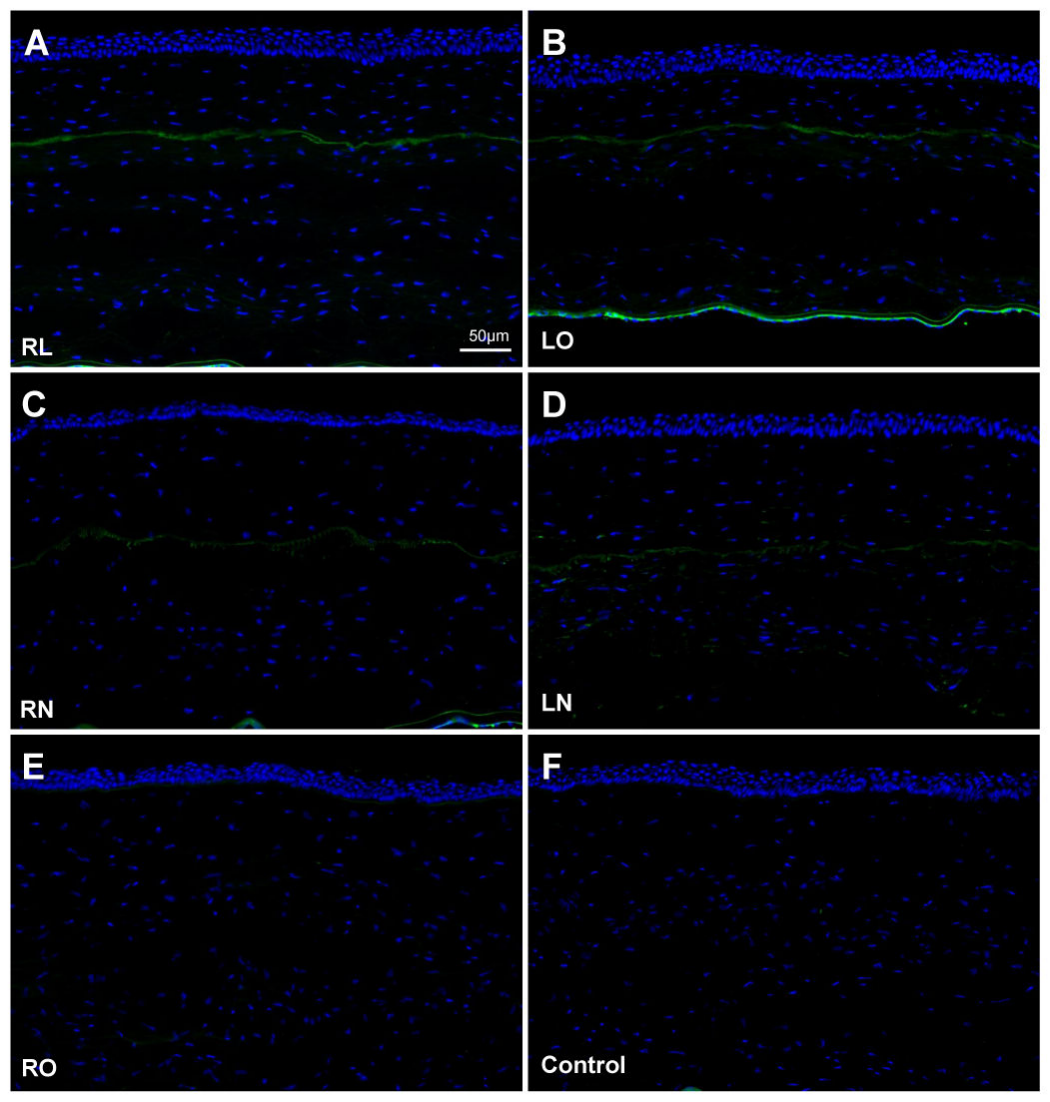
Wound healing reaction detected by fibronectin in the central cornea. (A) Fibronectin was expressed along the excimer laser ablation plane in corneas that underwent lenticule re-implantation and subsequent LASIK (RL group) and also in (B) corneas that underwent LASIK only (LO). (C) Relatively weaker fibronectin expression was observed along the laser incision plane in corneas that underwent lenticule re-implantation and subsequent creation of a LASIK flap, which was not lifted (RN), and also in (D) corneas that underwent creation of a LASIK flap, which was left intact (LN). (E) Fibronectin was absent in corneas that underwent lenticule re-implantation only (RO) and in (F) non-operated corneas (control). All images were captured at a magnification of 100x.

**Figure 7:**
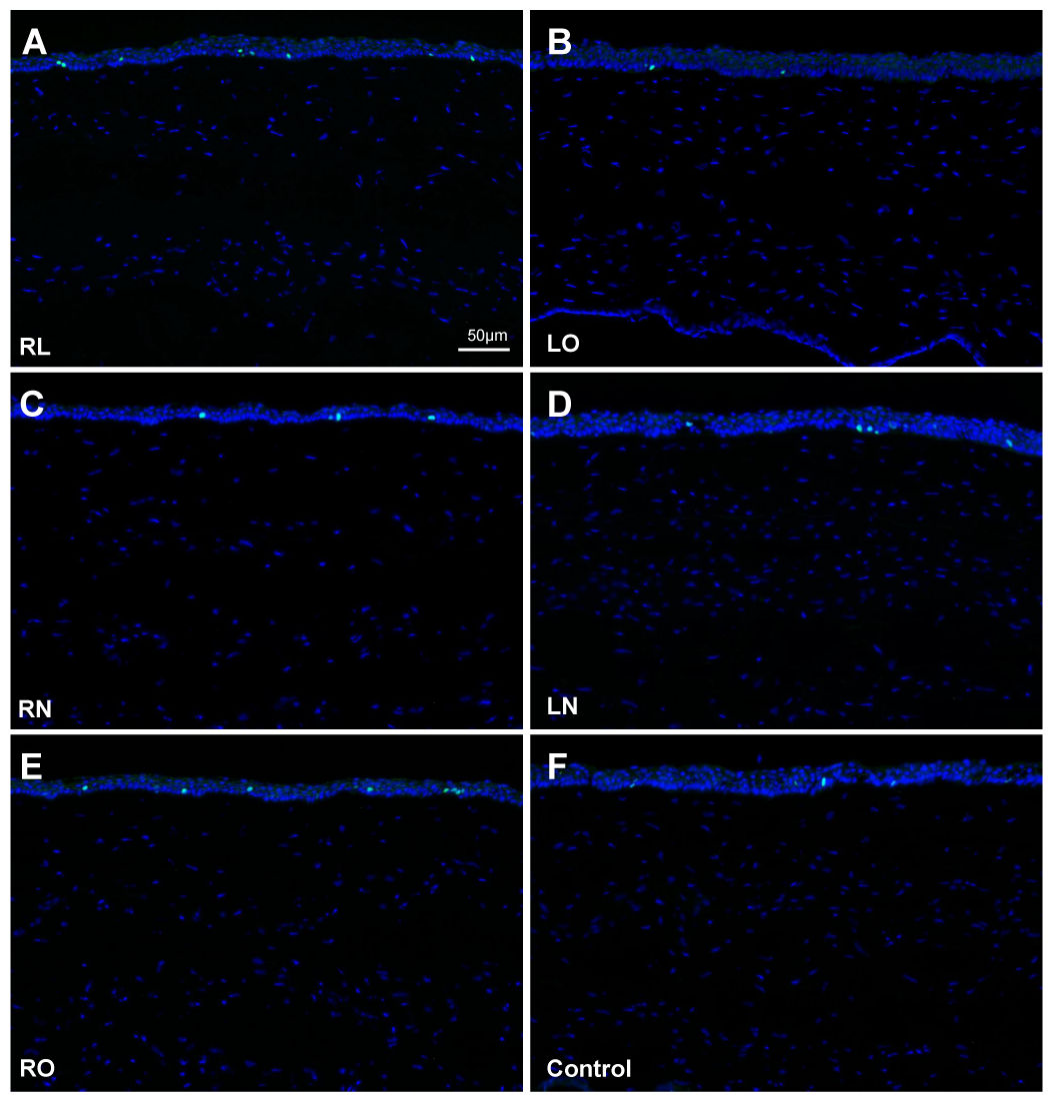
Proliferative cells in the central cornea detected by Ki-67 immunostaining. No Ki-67 expression was found within the stroma of (A) corneas that underwent lenticule re-implantation and subsequent LASIK (RL group), (B) corneas that underwent LASIK only (LO), (C) corneas that underwent lenticule re-implantation and subsequent creation of a LASIK flap, which was not lifted (RN), (D) corneas that underwent creation of a LASIK flap, which was left intact (LN), and also (E) corneas that underwent lenticule re-implantation only (RO), as well as in (F) non-operated corneas (control). Proliferative cells were predominantly found in the basal epithelial cells in all groups. All images were captured at a magnification of 100x.

Fibronectin was expressed along the excimer laser ablated plane in the central corneal stroma of the RL and LO groups (Figure 6A and B), with weaker expression along the non-lifted flap interface in the RN and LN groups (Figure 6C and D). No fibronectin was present 5 weeks after lenticule re-implantation (Figure 6E) and in control corneas (Figure 6F). No Ki-67-positive cells were detected in the central corneal stroma of all experimental groups and control corneas (Figure 7A-F). The proliferative cells were predominantly seen in the basal epithelial cells. 

**Figure 8:**
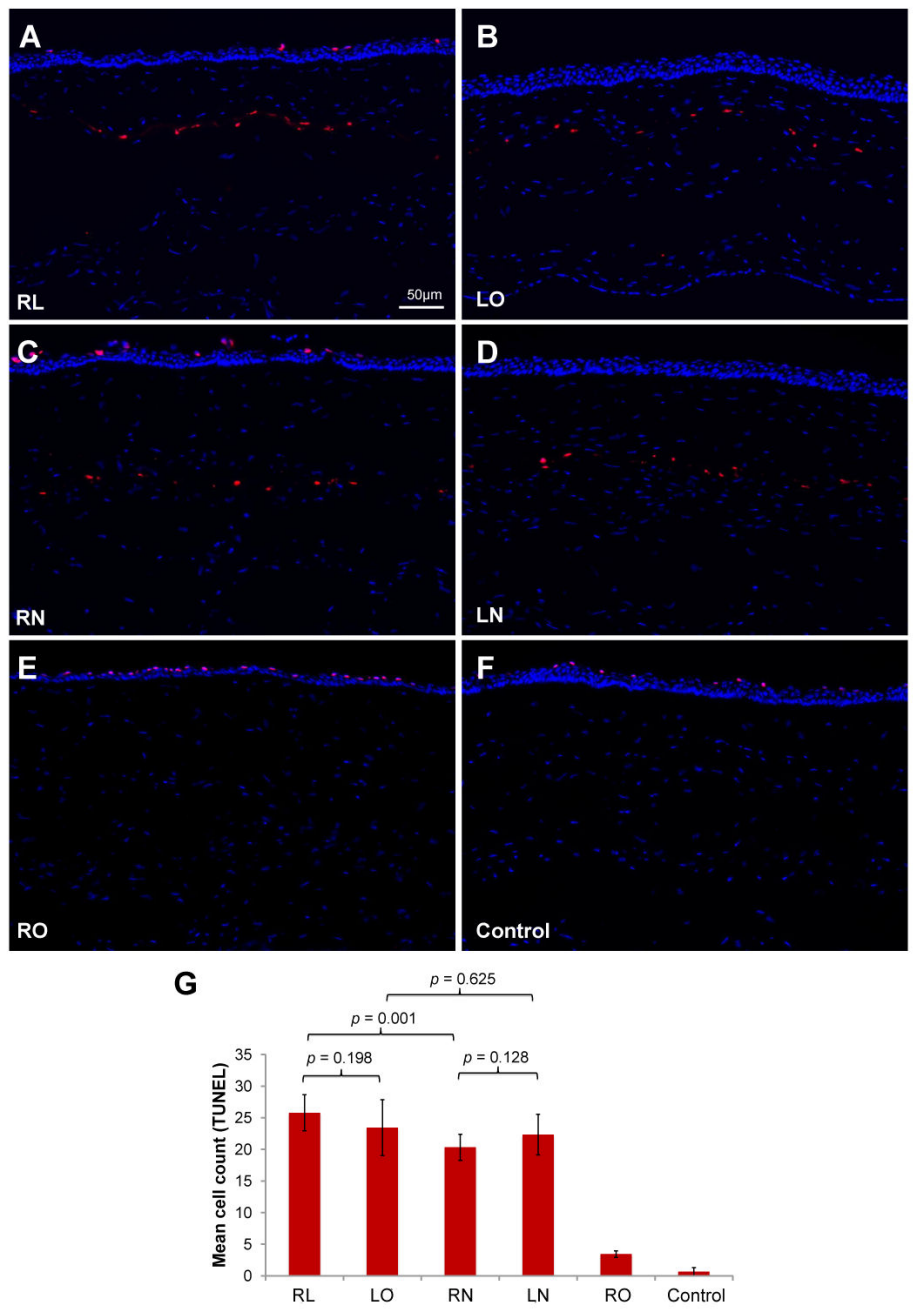
Apoptotic cells in the central cornea detected by TUNEL assay. (A) TUNEL-positive cells were seen along the excimer laser ablation plane in cornea that underwent lenticule re-implantation and subsequent LASIK (RL group) and also in (B) corneas that underwent LASIK only (LO). Relatively less apoptotic cells were observed in (C) corneas that underwent lenticule re-implantation and subsequent creation of a LASIK flap, which was not lifted (LN) and also in (D) corneas that underwent creation of a LASIK flap, which was left intact (LN). (E) No TUNEL-positive cells were detected in corneas that underwent lenticule re-implantation only (RO) and in (F) non-operated corneas (control). All images were captured at a magnification of 100x. (F) Mean cell counts in the central cornea stroma following TUNEL staining. Error bars represent standard deviation.

There were a significant number of TUNEL-positive cells in areas where laser ablation was performed in the RL and LO groups (Figure 8A and B). Apoptotic cells were also seen along the non-lifted flap interface in the RN and LN groups (Figure 8C and D). No cell death was observed within the corneal stroma of rabbits in the RO and control groups (Figure 8E and F). The mean TUNEL-positive cell count was tallied (Figure 8G) and no statistically significant differences were demonstrated between the RL and LO groups (P=0.198), and between RN and LN groups (P=0.128). No significant difference was noted between the LO and LN groups (P=0.625). However, as expected, there was a significant difference between the RL and RN groups (P=0.001).

## Discussion

LASIK has undergone tremendous advancements to achieve its current well-established safety and efficacy profile [2,24]. These include the integration of eye trackers, small spot scanning lasers, and wavefront-optimized/guided ablation technology [2]. Despite such improvements, LASIK, like all other previous forms of refractive laser correction, involves the irreversible removal of corneal tissue to achieve the required refractive correction. However, the introduction of ReLEx has possibly altered the refractive surgical landscape. ReLEx and its associated lenticule extraction techniques are the only theoretically reversible corneal refractive laser procedure to date [19,20]. The ability to reverse laser surgical procedures has several clinical applications. For instance, corneal stromal volume and biomechanical strength may be restored with either autologous or allogenic lenticule re-implantation in the treatment of various forms of keratectasia. Corneal stromal restoration also provides a unique opportunity to restore the myopic status in the non-dominant eye to previously low myopia, thereby resulting in presbyopic monovision. An alternative strategy for the management of presbyopia is the possibility of re-implanting the lenticule, which has been re-shaped into a graft with a smaller diameter and positive refractive power. In this scenario, the lenticule could act as a presbyopic intrastromal inlay.

The ability to achieve corneal volume restoration may also enable further refractive procedures to be performed. This is of particular significance in patients who have chosen to undergo laser refractive correction earlier in their life. With the inevitable development of presbyopia, refractive laser treatments, such as monovision LASIK [22] and presbyLASIK [25] are possible following re-implantation. Although monocular implantation of a presbyopic intrastromal inlay, such as the Kamra (AcuFocus, Irvine, CA) or PresbyLens (ReVision Optics, Lake Forest, CA), could be a viable option for this group of patients without re-implantation, biocompatibility related complications can potentially arise from using non-biological implants, which include alterations in tear film thickness and corneal topography [26], corneal erosions [27], and peri-inlay deposits [28].

Following ReLEx, patients who have undergone stromal volume restoration via autologous lenticule re-implantation could potentially undergo a second refractive laser procedure in the management of their presbyopia or refinement of any residual refractive error. We acknowledge that surface ablation (PRK) would be another feasible option but the aim of our experiments was to assess the effect of subsequent excimer laser ablation on the lenticule itself. Our study explored the feasibility of performing a secondary laser procedure, LASIK, in a presbyopic patient who had previously undergone myopic refractive laser surgery by utilizing a rabbit model of SMILE. The animal model in this study was allocated to different treatment groups: (i) lenticule re-implantation and subsequent LASIK procedure; (ii) lenticule re-implantation and subsequent LASIK flap creation, which was left un-lifted; (iii) lenticule re-implantation only; (iv) LASIK procedure alone; (v) LASIK flap creation alone, which was left un-lifted; and (vi) un-operated control corneas, in order to provide an understanding of the corneal tissue response to different aspects of the procedure. 

In our previous studies [19,20], the refractive lenticule was re-implanted by overlying the lenticule on the exposed stromal bed after the flap (created by FLEx) was re-lifted. Here, lenticule re-implantation was performed through the previously created keyhole incision following the initial SMILE procedure. The current technique resembles keratophakia which was first described by Barraquer [29], where donor corneal tissue was manually lathed to a desired refractive power and inserted into an intrastromal pocket. The failure to widely adopt this technique clinically resulted from various post-operative complications including interface scarring between the lenticule and patient’s cornea, and unpredictable astigmatic outcomes. In contrast, the technique of refractive lenticule re-implantation described here has significant advantages in terms of laser-accurate-lamellar cut and -refractive correction. In addition, the low-energy FSL system employed to perform ReLEx does not induce excessive cell death and inflammation [14], which may be beneficial for the healing of the lenticular interfacial wound.

Similar to re-implantation following FLEx [19,20], corneal haze and inflammatory cells could be seen at the anterior and posterior borders of the re-implanted lenticule on in vivo confocal microscopy early after the surgery. These features resolved over the 5-week follow-up period. This observation was confirmed by slit lamp examination, where progressive improvement in corneal clarity was observed. Immunohistochemistry staining of fibronectin, CD11b, Ki-67 and TUNEL showed no difference from control corneas 5 weeks after lenticule re-implantation.

Since the keratophakia technique described here is an additive procedure, it provides the unique opportunity for performing further laser refractive surgery. The rabbit corneas were initially subjected to a -6.00D spherical correction with SMILE and reversal of this myopic correction 14 days later. Five weeks after lenticule re-implantation, a LASIK flap was created just anterior to the lenticular anterior interface and a -5.00D spherical correction was performed with an excimer laser to simulate a monovision correction by creating a 1.00D difference between both eyes. An anomaly that was found prior to performing LASIK was the depth of the anterior interface of the lenticule, which was measured at approximately 90 µm on AS-OCT in all cases (n=6) rather than the equivalent depth of the SMILE corneal cap at 130 µm. Therefore, we had to create a thin flap with a thickness of 80 µm to avoid intersecting the anterior interface of the lenticule. The reason behind this discrepancy between the depth of the programmed cap cut and anterior interface of the reimplanted lenticule may be that the addition of the lenticule had expanded the intrastromal pocket volume, thereby altering the anterior stromal architecture. Interestingly, a similar phenomenon was observed 1 month following lenticule re-implantation in our rabbit model of FLEx [19], but not in the monkey model of FLEx [20]. The shifting of the anterior lenticular interface between pre- and post-reimplantation may have occurred due to differences in response to the lenticule insertion between species. Differences between the corneal anatomy of rabbits and primates (rabbits have a poorly-developed Bowman’s membrane compared to humans [30]) and biomechanical properties (human corneal stroma has been demonstrated to posses a stronger interlamellar binding compared to rabbit stroma [31,32]) may also have contributed to the different findings between animal models. 

Intrastromal irregularities on slit lamp examination, corresponding to elevated anterior interface reflectivity levels observed on in vivo confocal microscopy images were noted in the RL group one-day post-LASIK. Quantitatively, the results were comparable (P = 0.310) to in vivo confocal microscopy findings in corneas that had undergone LASIK only. In addition, the RL and LO groups showed similar patterns and expression levels of fibronectin, as well as similar number of inflammatory cells (P = 0.304) and apoptotic cells (P = 0.198). In the RN and LN groups, we found a relatively weaker expression of fibronectin and lower number of inflammatory cells. The disparity in the wound healing and inflammatory responses can be attributed to the differences in corneal tissue damage inflicted during tissue ablation by the excimer laser compared to tissue incision by the FSL [14]. These results suggest that there are no significant differences in the corneal tissue response and development of early post-operative corneal haze after performing LASIK on corneas that have previously undergone SMILE and subsequent lenticule re-implantation, in comparison with corneas that have not previously undergone any previous refractive surgical procedures. 

The SMILE lenticule re-implantation technique, which involves the insertion and placement of the lenticule through a keyhole incision beneath then corneal cap, obviously obviates much of the inflammatory and wound healing responses, as well as avoids compromising the epithelial and basement membrane integrity with the absence of flap side cut. These advantages improve graft-host tissue integration and may potentially reduce the duration required for corneal recovery, allowing the secondary laser refractive surgery to be performed 1-2 months after lenticule re-implantation. 

For cryopreservation of the lenticule, dimethyl sulfoxide (DMSO) was used as a cryoprotectant to prevent cellular damage during the freezing in liquid nitrogen. Tissue edema was present following 2-weeks of cryopreservation. The edematous lenticule expanded the intrastromal pocket and a small gap was noted at the small incision site following insertion. This prompted us to place a suture to close the gap to prevent epithelial ingrowth and possible diffuse lamellar keratitis. As aforementioned, expansion of the intrastromal pocket may also have caused shifting of the anterior border of the reimplanted lenticule, which could alter the corneal anterior architecture and potentially result in under- or over-correction of the refractive error. The unpredictable refractive outcomes after lenticule re-implantation may not be critical in the treatment method described in this study, because subsequent LASIK would be able to correct the refractive regression so that monovision can be accurately achieved. Nevertheless, other cryopreservatives and methods of freeze-drying lenticules are now being explored in our laboratory in an attempt to produce a more stable graft in terms of thickness and clarity.

We attempted to measure the refractive spherical error and astigmatism 1 day post-LASIK to assess the efficacy of the technique described in the study, but the measurements appeared inconsistent or inaccurate. This could be due to the early post-surgical wound healing responses and changes in corneal biomechanics. A follow-up study is now under way in our lab to study the efficacy of the technique in a longer term using an experimental primate model and the changes in refractive state will then be addressed.

In conclusion, we have demonstrated that LASIK can be performed following SMILE lenticule re-implantation, in creating presbyopic monovision in patients who have previously undergone SMILE for the correction of myopia. The corneal tissue responses elicited after performing LASIK on corneas that have previously undergone SMILE and subsequent lenticule re-implantation are no different from the responses evoked in corneas that have not previously undergone any refractive surgical procedures. Moreover, our study indicates that re-implantation of the lenticule in the patient’s cornea that has previously undergone a laser refractive surgery can provide a unique opportunity for the patient to undergo further presbyopic corrective surgery, not limited to monovision LASIK, but also other commercially available presbyopic LASIK programmes, such as Supracor (Bausch + Lomb), Presbybond Laser Blended Vision (Carl Zeiss Meditec), PresbyMAX (Schwind, Kleinostheim, Germany) or PresbyLASIK (AMO, Santa Ana, CA).

## References

[1] American Academy of Ophthalmology (2008) Is LASIK for me? A patient's guide to refractive surgery. Available: http://www.geteyesmart.org/eyesmart/glasses-contacts-lasik/upload/LASIK-patient-guide.pdf. Accessed 15 August 2013

[2] Maldonado MJ, Nieto JC, PiñEro DP (2008) Advances in technologies for laser-assisted in situ keratomileusis (LASIK). Surgery - Expert Rev Med Devices 5: 209-229. doi:10.1586/17434440.5.2.209. 18331182

[3] Skevas C, Katz T, Wagenfeld L, Richard G, Linke S (2013) Subjective pain, visual recovery and visual quality after LASIK, EpiLASIK (flap off) and APRK — a consecutive, non-randomized study. Graefes Arch Clin Exp Ophthalmol 251: 1-9. PubMed: 22527326. 10.1007/s00417-012-2181-7 23096124

[4] Fares U, Otri AM, Al-Aqaba MA, Faraj L, Dua HS (2012) Wavefront-optimized excimer laser in situ keratomileusis for myopia and myopic astigmatism: Refractive outcomes and corneal densitometry. J Cataract Refract Surg 38: 2131-2138. doi:10.1016/j.jcrs.2012.07.041. PubMed: 23084157. 23084157

[5] Kezirian GM, Stonecipher KG (2004) Comparison of the IntraLase femtosecond laser and mechanical keratomes for laser in situ keratomileusis. J Cataract Refract Surg 30: 804-811. doi:10.1016/j.jcrs.2003.10.026. PubMed: 15093642. 15093642

[6] Nordan LT, Slade SG, Baker RN, Suarez C, Juhasz T et al. (2003) Femtosecond laser flap creation for laser in situ keratomileusis: six-month follow-up of initial U.S. clinical series. J Refract Surg 19: 8-14. PubMed: 12553599. 12553599 10.3928/1081-597X-20030101-03

[7] Montés-Micó R, Rodríguez-Galietero A, Alió JL (2007) Femtosecond laser versus mechanical keratome LASIK for myopia. Ophthalmology 114: 62-68. doi:10.1016/j.ophtha.2006.07.019. PubMed: 17070593. 17070593

[8] Calvo R, McLaren JW, Hodge DO, Bourne WM, Patel SV (2010) Corneal aberrations and visual acuity after laser in situ keratomileusis: femtosecond laser versus mechanical microkeratome. Am J Ophthalmol 149: 785-793. doi:10.1016/j.ajo.2009.12.023. PubMed: 20227675. 20227675 PMC2856792

[9] Ang RT, Dartt DA, Tsubota K (2001) Dry eye after refractive surgery. Curr Opin Ophthalmol 12: 318-322. doi:10.1097/00055735-200108000-00013. PubMed: 11507347. 11507347

[10] Stonecipher KG, Dishler JG, Ignacio TS, Binder PS (2006) Transient light sensitivity after femtosecond laser flap creation: clinical findings and management. J Cataract Refract Surg 32: 91-94. doi:10.1016/j.jcrs.2005.11.015. PubMed: 16516785. 16516785

[11] Lifshitz T, Levy J, Klemperer I, Levinger S (2005) Late bilateral keratectasia after LASIK in a low myopic patient. J Refract Surg 21: 494-496. PubMed: 16209448. 16209448 10.3928/1081-597X-20050901-12

[12] Kullman G, Pineda R 2nd (2010) Alternative applications of the femtosecond laser in ophthalmology. Semin Ophthalmol 25: 256-264. doi:10.3109/08820538.2010.518507. PubMed: 21091009. 21091009

[13] Juhasz T, Kastis GA, Suárez C, Bor Z, Bron WE (1996) Time-resolved observations of shock waves and cavitation bubbles generated by femtosecond laser pulses in corneal tissue and water. Lasers Surg Med 19: 23-31. doi:10.1002/(SICI)1096-9101(1996)19:1. PubMed: 8836993. 8836993

[14] Riau AK, Angunawela RI, Chaurasia SS, Lee WS, Tan DT et al. (2011) Early corneal wound healing and inflammatory responses after refractive lenticule extraction (ReLEx). Invest Ophthalmol Vis Sci 52: 6213-6221. doi:10.1167/iovs.11-7439. PubMed: 21666235. 21666235

[15] Ang M, Chaurasia SS, Angunawela RI, Poh R, Riau A et al. (2012) Femtosecond lenticule extraction (FLEx): clinical results, interface evaluation, and intraocular pressure variation. Invest Ophthalmol Vis Sci 53: 1414-1421. doi:10.1167/iovs.11-8808. PubMed: 22323464. 22323464

[16] Blum M, Kunert K, Schröder M, Sekundo W (2010) Femtosecond lenticule extraction for the correction of myopia: preliminary 6-month results. Graefes Arch Clin Exp Ophthalmol 248: 1019-1027. doi:10.1007/s00417-009-1293-1. PubMed: 20130899. 20130899

[17] Sekundo W, Kunert KS, Blum M (2011) Small incision corneal refractive surgery using the small incision lenticule extraction (SMILE) procedure for the correction of myopia and myopic astigmatism: results of a 6 month prospective study. Br J Ophthalmol 95: 335-339. doi:10.1136/bjo.2009.174284. PubMed: 20601657. 20601657

[18] Vestergaard AH, Grønbech KT, Grauslund J, Ivarsen AR, Hjortdal JO (2013) Subbasal nerve morphology, corneal sensation, and tear film evaluation after refractive femtosecond laser lenticule extraction. Graefes Arch Clin Exp Ophthalmol (. (2013)) PubMed: 23793872. 10.1007/s00417-013-2400-x 23793872

[19] Angunawela RI, Riau AK, Chaurasia SS, Tan DT, Mehta JS (2012) Refractive lenticule re-implantation after myopic ReLEx: a feasibility study of stromal restoration after refractive surgery in a rabbit model. Invest Ophthalmol Vis Sci 53: 4975-4985. doi:10.1167/iovs.12-10170. PubMed: 22743323. 22743323

[20] Riau AK, Angunawela RI, Chaurasia SS, Lee WS, Tan DT et al. (2013) Reversible femtosecond laser-assisted myopia correction: A non-human primate study of lenticule reimplantation after refractive lenticule extraction. PLOS ONE 8: e67058. doi:10.1371/journal.pone.0067058. PubMed: 23826194. 23826194 PMC3691223

[21] Mohamed-Noriega K, Toh KP, Poh R, Balehosur D, Riau A et al. (2011) Cornea lenticule viability and structural integrity after refractive lenticule extraction (ReLEx) and cryopreservation. Mol Vis 217: 3437-3449. PubMed: 22219639. PMC3249438 22219639

[22] Goldberg DB (2001) Laser in situ keratomileusis monovision. J Cataract Refract Surg 27: 1449-1455. doi:10.1016/S0886-3350(01)01001-X. PubMed: 11566531. 11566531

[23] Riau AK, Angunawela RI, Chaurasia SS, Tan DT, Mehta JS (2012) Effect of different femtosecond laser-firing patterns on collagen disruption during refractive lenticule extraction. J Cataract Refract Surg 38: 1467-1475. doi:10.1016/j.jcrs.2012.03.037. PubMed: 22814054. 22814054

[24] Lawless M, Hodge C (2013) Lasik. Int Ophthalmol Clin 53: 111-128. doi:10.1097/IIO.0b013e318271346e. PubMed: 23221889. 23221889

[25] Alió JL, Chaubard JJ, Caliz A, Sala E, Patel S (2006) Correction of presbyopia by technovision central multifocal LASIK (presbyLASIK). J Refract Surg 22: 453-460. PubMed: 16722483. 16722483 10.3928/1081-597X-20060501-06

[26] Dexl AK, Ruckhofer J, Riha W, Hohensinn M, Rueckl T et al. (2011) Central and peripheral corneal iron deposits after implantation of a small-aperture corneal inlay for correction of presbyopia. J Refract Surg 27: 876-880. doi:10.3928/1081597X-20110802-02. PubMed: 21815605. 21815605

[27] Evans MD, Prakasam RK, Vaddavalli PK, Hughes TC, Knower W et al. (2011) A perfluoropolyether corneal inlay for the correction of refractive error. Biomaterials 32: 3158-3165. doi:10.1016/j.biomaterials.2011.01.047. PubMed: 21306775. 21306775

[28] Mulet ME, Alio JL, Knorz MC (2009) Hydrogel intracorneal inlays for the correction of hyperopia: outcomes and complications after 5 years of follow-up. Ophthalmology 116: 1455-1460. doi:10.1016/j.ophtha.2009.05.019. PubMed: 19651310. 19651310

[29] Barraquer JI (1972) Keratophakia. Trans Ophthalmol Soc U K 92: 499-516. PubMed: 4515533. 4515533

[30] Gipson IK, Spurr-Michaud SJ, Tisdale AS (1987) Anchoring fibrils form a complex network in human and rabbit cornea. Invest Ophthalmol Vis Sci 28: 212-220. PubMed: 8591898. 8591898

[31] Maurice DM, Monroe F (1990) Cohesive strength of corneal lamellae. Exp Eye Res 50: 59-63. doi:10.1016/0014-4835(90)90011-I. PubMed: 2307196. 2307196

[32] Maurice DM (1988) Mechanics of the cornea. In: Cavanagh HD The Cornea: Transactions of the World Congress on the Cornea III. New York Raven Press pp. 187-193.

